# Datasets for mapping pastoralist movement patterns and risk zones of Rift Valley fever occurrence

**DOI:** 10.1016/j.dib.2017.11.097

**Published:** 2017-12-06

**Authors:** Gladys Mosomtai, Magnus Evander, Charles Mundia, Per Sandström, Clas Ahlm, Osama Ahmed Hassan, Olivia Wesula Lwande, Moses K. Gachari, Tobias Landmann, Rosemary Sang

**Affiliations:** a International Centre of Insect Physiology and Ecology, P. O. Box 30772-00100, Nairobi, Kenya; b Department of Clinical Microbiology, Virology, Umeå University, 901 85 Umeå, Sweden; c Department of Forest Resource Management, Swedish University of Agricultural Sciences, Faculty of Forest Sciences, 901 83 Umeå, Sweden; d Department of Clinical Microbiology, Infectious Diseases, Umeå University, 901 85 Umeå, Sweden; e Institute of Geomatics, GIS & Remote Sensing, Dedan Kimathi University of Technology, P.O. Box 657-10100, Nyeri, Kenya

**Keywords:** Home range estimation, Vector distribution, Rift Valley fever

## Abstract

Rift Valley fever (RVF) is a zoonotic disease affecting humans and animals. It is caused by RVF virus transmitted primarily by *Aedes* mosquitoes. The data presented in this article propose environmental layers suitable for mapping RVF vector habitat zones and livestock migratory routes. Using species distribution modelling, we used RVF vector occurrence data sampled along livestock migratory routes to identify suitable vector habitats within the study region which is located in the central and the north-eastern part of Kenya. Eleven herds monitored with GPS collars were used to estimate cattle utilization distribution patterns. We used kernel density estimator to produce utilization contours where the 0.5 percentile represents core grazing areas and the 0.99 percentile represents the entire home range. The home ranges were overlaid on the vector suitability map to identify risks zones for possible RVF exposure. Assimilating high spatial and temporal livestock movement and vector distribution datasets generates new knowledge in understanding RVF epidemiology and generates spatially explicit risk maps. The results can be used to guide vector control and vaccination strategies for better disease control.

**Specifications Table**

**Table:** 

Subject area	*Spatial epidemiology*
More specific subject area	*Disease mapping, movement ecology.*
Type of data	*Raster and vector (point and polygon) data*
How data was acquired	*Normalized Difference Vegetation Index (NDVI) from 250* *m MOD 13Q product, Evapotranspiration from 1* *km MOD 16*[1]*, Topographic wetness index (TWI) from 90* *m SRTM DEM, soil types from 1:50,000 soil map from Soil survey of Kenya*[2]*, Bioclimatic variables from 1* *km AfriClim*[3]*, mosquito vectors occurrence data sampled using Garmin Etrex 20x GPS - Model 010–01508-00, cattle trajectory from Followit Iridium collars.*
Data format	*Raw, analyzed (tiffs, ascii & shp.)*
Experimental factors	*Extraction of seasonality parameters from NDVI, reducing dimensionality, test for collinearity of variables.*
Experimental features	*TIMESAT*[4]*was used to extract seasonality parameters from NDVI time series data spanning from 2001 to 2015.*
	*Principal component Analysis was used to reduce data dimensionality of satellite-derived evapotranspiration for 2001–2013*
	*Variance inflation factors was applied on AfriClim data.*
Data source location	*Lies within the bounding box of Latitude E36.724*° *Longitude N2.2820*° *and Longitude E41.6921*° *Latitude S3.2230*°*, which traverses Isiolo, Garissa, Tana River and Lamu counties in Kenya.*
Data accessibility	*Provided in this article*

**Value of the data**

• Vegetation seasonality, topography, soil types and climatic data can be used to understand ecological characteristics of mosquito habitats as a factor for RVF propagation.
• Livestock movement patterns can be used to explore the role of animal movement in RVF propagation.
• The datasets can be integrated and used to identify risk zones for RVF hence, improve the effectiveness of intervention strategies against the disease.

## 1 Data

**Fig. 1:**
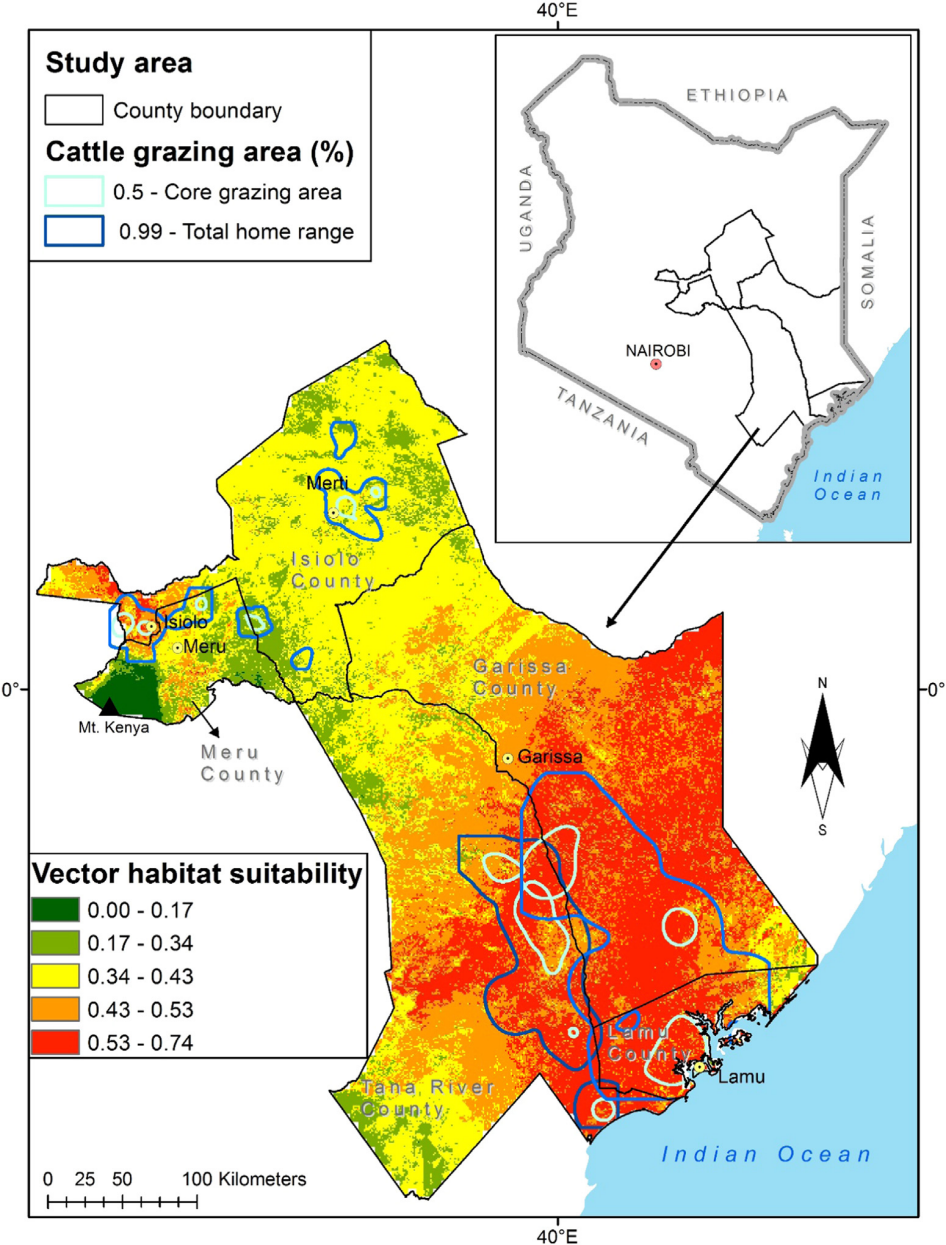
Integrated vector habitat suitability and cattle home range map. Reddish shades represents suitable vector habitat conditions while green represents non-suitable habitats for RVF vectors. Cattle grazing areas are shown as curved lines whereby 0.5 represents the core grazing areas and the 0.99 represents the entire home range.

**Fig. 2:**
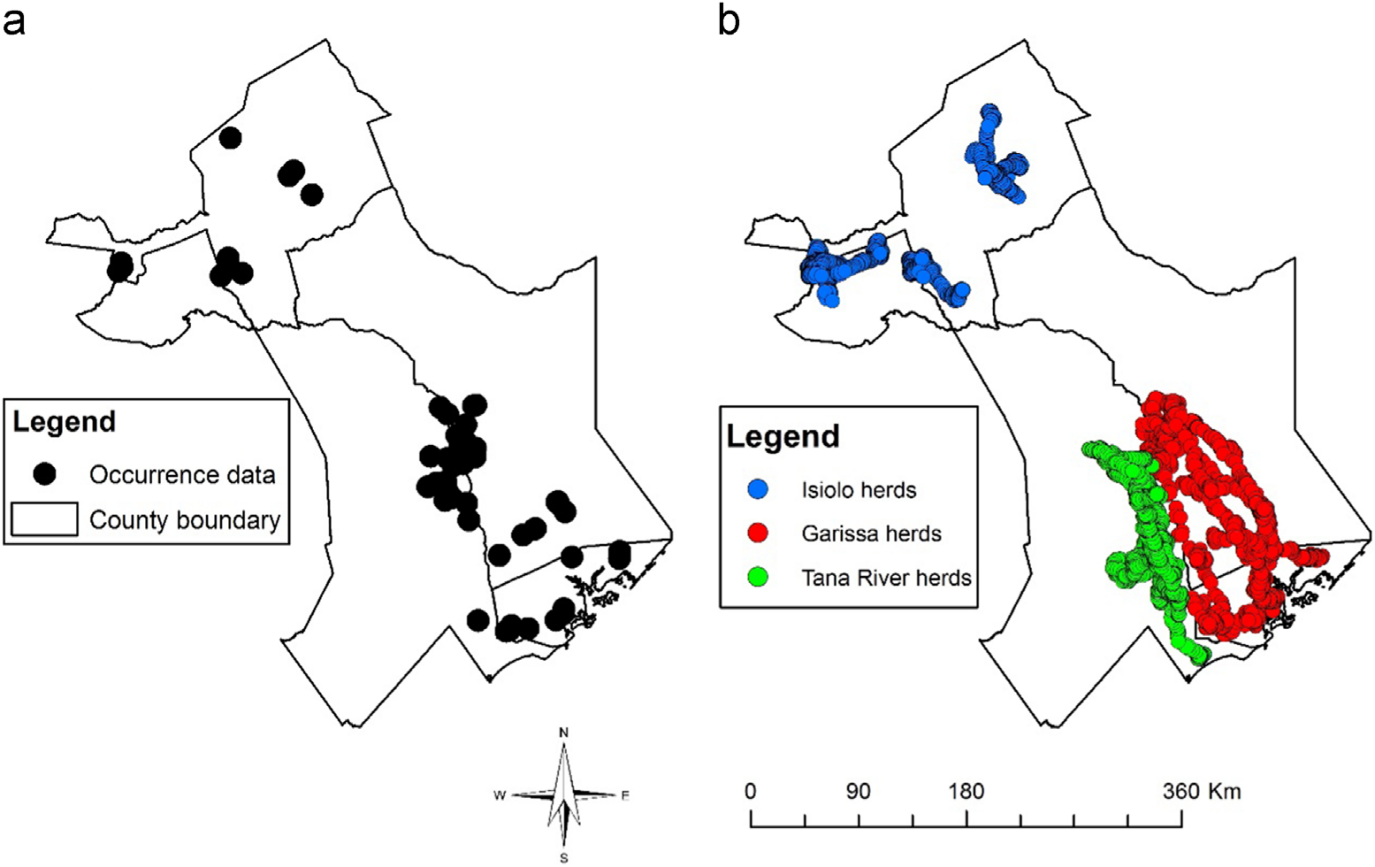
Map showing a) sampled RVF vectors along cattle migratory routes, b) migratory routes of collared herds.

**Fig. 3:**
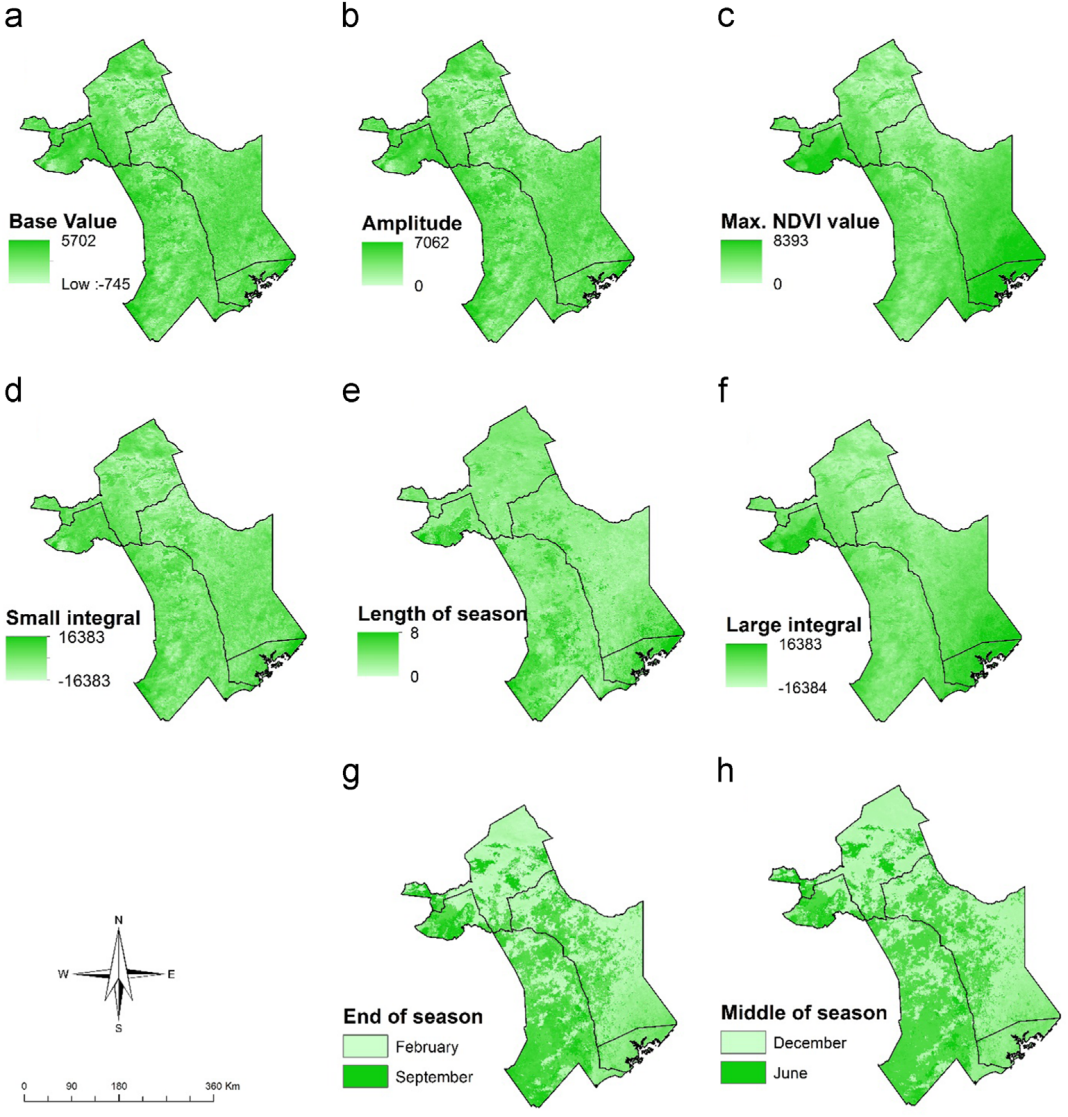
Maps of vegetation seasonality parameters extracted from TIMESAT; a) Base NDVI value, b) amplitude, c) Maximum NDVI value in the season, d) Small integral value, e) Length of season (months), f) Large integral value, g) end of season and h) middle of season.

**Fig. 4:**
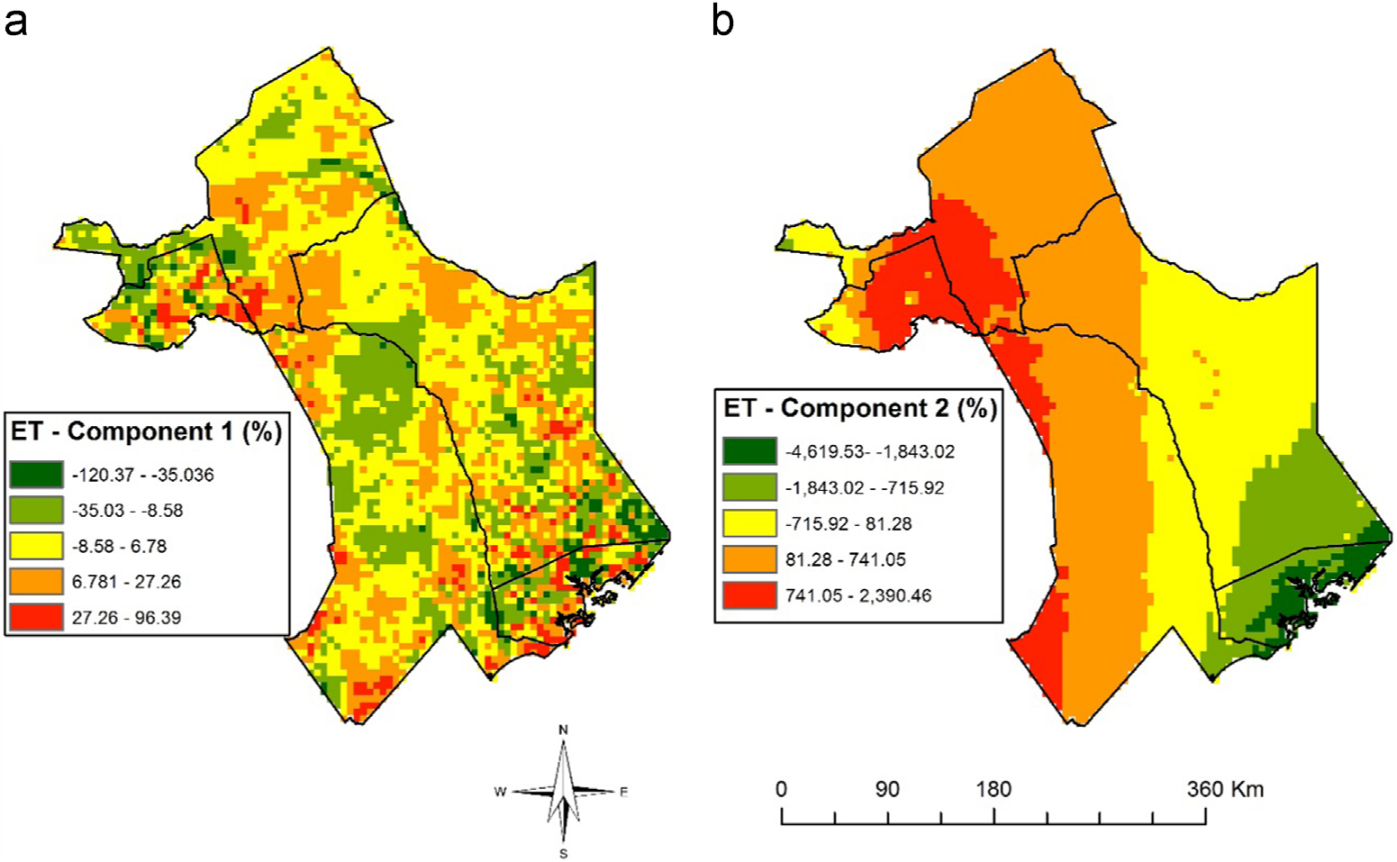
Maps showing the first (a) and second (b) principal components for evapotranspiration data. The legend represents the amount of variance in the data (eigenvalues) with green shade representing low variance while red shade represents high variance in each component.

**Fig. 5:**
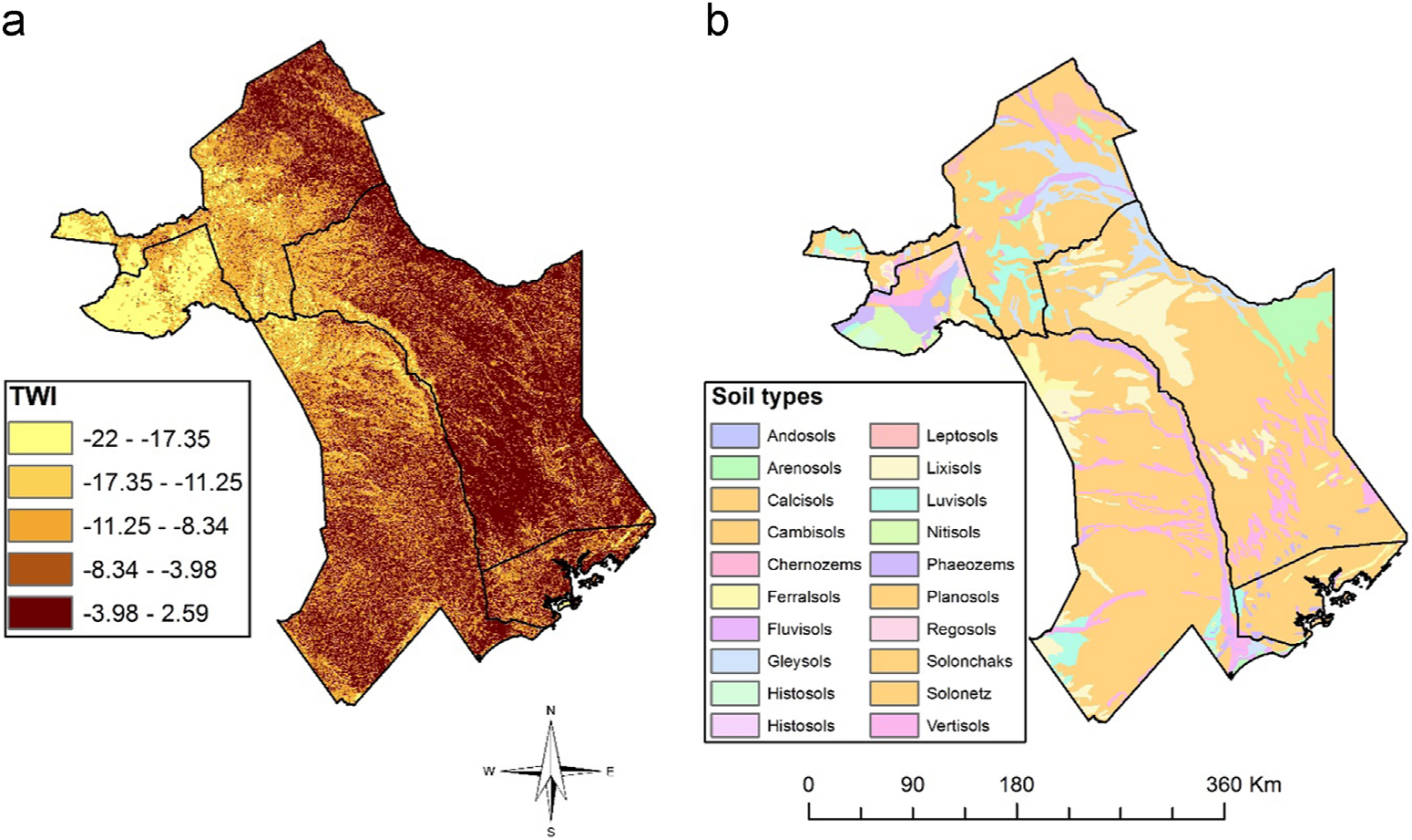
Maps showing topographic wetness index (TWI) (a) and soil types (b) of the study area. Deep brown colour in TWI represents high water saturation areas such as plains and *dambos* whereas light brown shades represent higher ridges and hills with no water saturation.

**Fig. 6:**
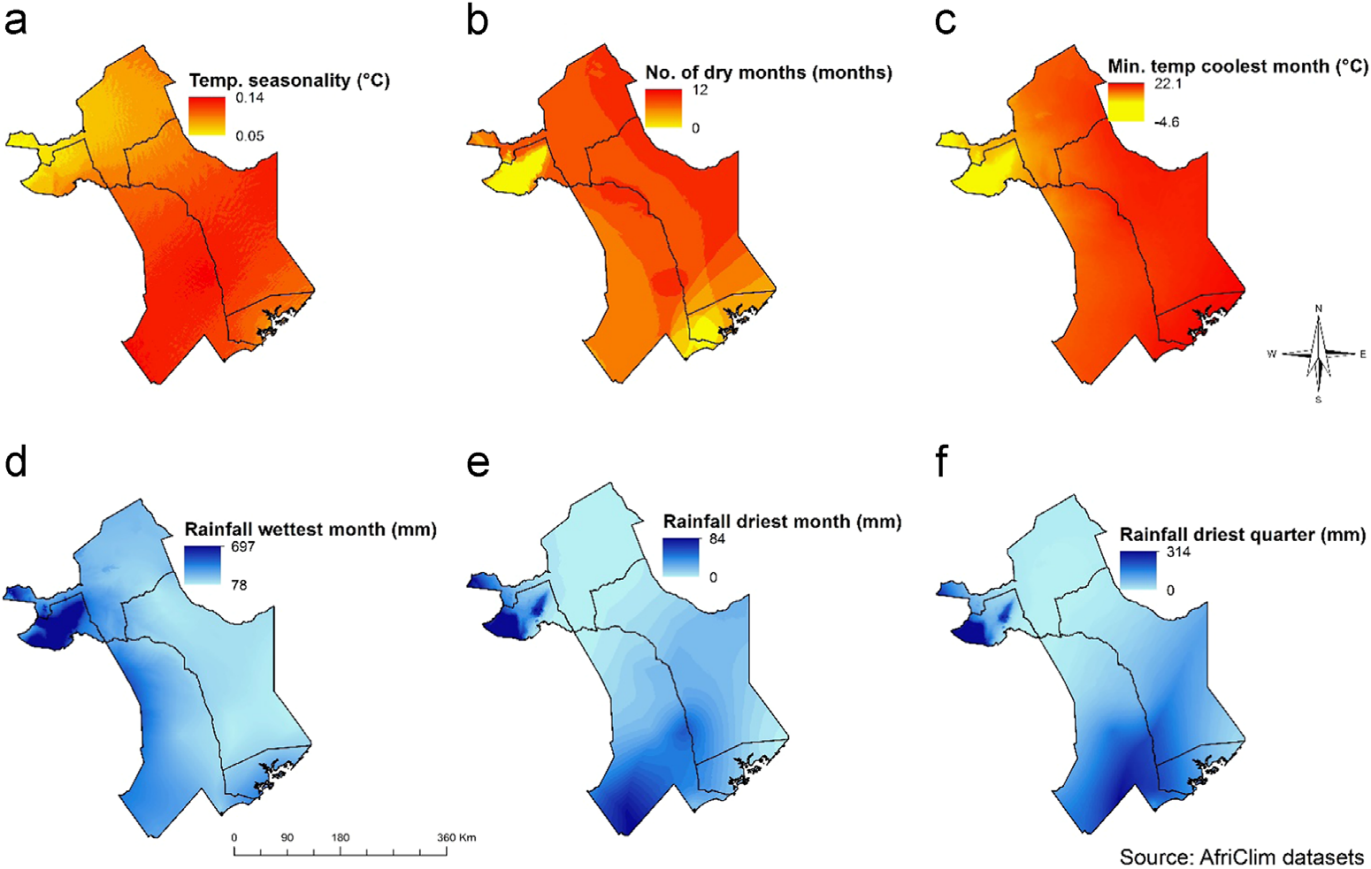
Map showing climatic characteristics of the study area; a) Temperature seasonality (°C), b) Number of dry months (months), c) Minimum temperature coolest month (°C), d) Rainfall wettest month (mm), e) Rainfall driest month (mm) and f) Rainfall driest quarter (mm).

This article presents datasets used to map exposure of pastoralist to RVF vectors along their migratory routes. Fig. 1 shows habitat suitability for RVF vectors overlaid with livestock grazing areas. Fig. 2a shows the location of sampled RVF vectors while Fig. 2b shows the trajectory of the collared herds. Fig. 3, Fig. 4, Fig. 5, Fig. 6 shows the environmental characteristics of the study area.

## 2 Experimental design, materials and methods

### 2.1 Cattle movement data

Table 1 shows the summary of the datasets used in the study. Fig. 2b shows cattle movement data obtained from 2012 to 2016 from 11 collared herds from Garissa, Tana River and Isiolo counties herein referred to as Garissa, Tana River and Isiolo herds respectively. We collared six Garissa herds between September 2012 and June 2014 while two Tana River and three Isiolo herds were collared from August 2013 to December 2016. The temporal resolution for transmission was after every one hour during the day i.e. twelve GPS location per herd between 6am and 6pm. However, there were several times when the collars failed to transmit because the animals were either out of range of the satellites or when the battery life ended.

**Table 1:** Summary of data sources.

Description	Data	Data formats	Source	Period	Resolution
Cattle movement	GPS collars	Vector (raw)	11 Herds	Sep 2012 to Jul 2016	1 hour fixes
Mosquito sampling	Lat, long	Vector (raw)	GPS	Apr/Dec 2012–2015	Long and short rains
Environmental layers	Evapotranspiration	Raster (processed)	MOD 16	2012–2015	1 km
	Soil type	Raster (processed)	Soil Survey of Kenya	Revised 1997	1:50, 000
	Elevation	Raster (processed)	USGS	N/A	30 m
	NDVI	Raster (processed)	University of Natural Resources and Life Science, Vienna	2001–2015	250 m
	Africlim	Raster (processed)	The university of York	1961–1990	1 km

### 2.2 Mosquito sampling

[5] and [6] articulate the procedure in which mosquito sampling was done. In both studies, approximately over 100,000 mosquitoes were sampled belonging to six genera namely; *Aedes, Anopheles, Mansonia, Culex, Aedeomyia and Coquillettidia*. Sampling was done during long (March, April, May) and short (October, November, December) rains and each sampling site was considered an occurrence point for species distribution modelling as shown in Fig. 2a

## 3 Environmental layers

We downloaded pre-processed 16-day NDVI and monthly MOD16 Evapotranspiration (ET) time series data for 2001–2015 from University of Natural Resources and Life Science, Vienna portal [7] and USGS data portal respectively [1]. Fig. 5b shows the soil type map obtained from the Kenya Soil Survey dataset while elevation data from 90 m Digital Elevation Model (DEM) from the Shuttle Radar Topographic Mission (SRTM) was obtained from USGS data portal. We also downloaded current climatic conditions from 1 km AfriClim datasets from The University of York portal as shown in Fig. 6 [3].

## 4 Methods

**Fig. 7:**
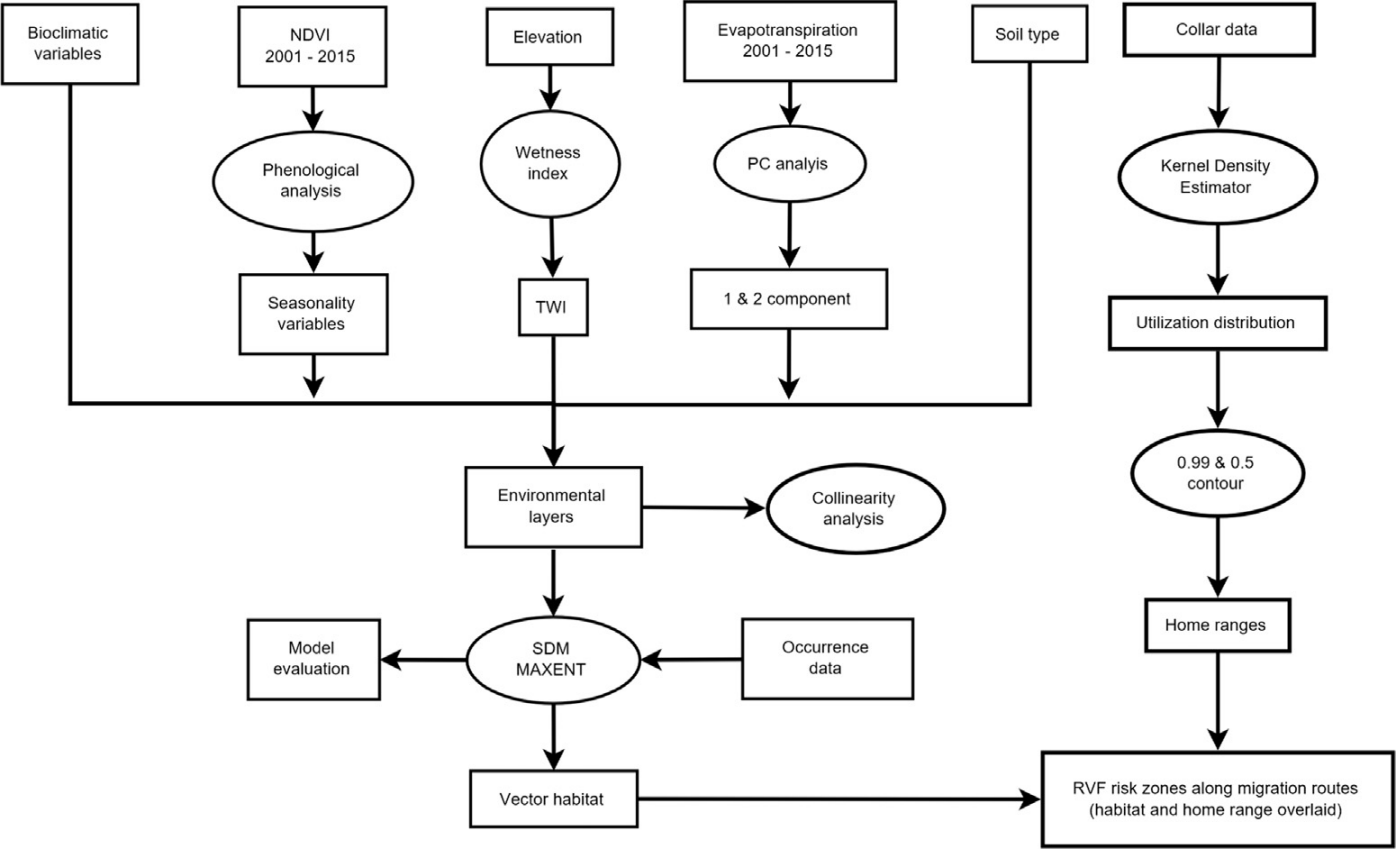
Flowchart showing the included variables and methods.

The data variables and methods are summarized in Fig. 7. The vegetation seasonality parameters shown in Fig. 3 were extracted from NDVI time series using TIMESAT [4]. A description for the meaning of each seasonality parameter extracted is provided by Jönsson and Eklundh [4]. We conducted a principle component analysis on ET time-series to obtain the data shown in Fig. 4. This reduced data dimensionality and maximized data variability over the observation period by extracting the underlying data structure [8], [9]. We extracted TWI from 90 m DEM data as shown in Fig. 5a using SAGA GIS to identify steadiness of wetness of the study area [10], [11]. Steadiness of wetness of an area is defined by the contribution the slope and the upstream region has in influencing its ability/capacity of retaining water in any particular time [12]. We aggregated seasonality parameters, ET components, TWI, soil type and AfriClim herein referred to as environmental layers (Fig. 7) and tested for multi-collinearity using Variance Inflation Factors (VIF) before using them for further analysis in species distribution modelling.

We used species distribution modelling technique to map vector habitat suitability. This was achieved by associating the occurrence data with environmental layers (Fig. 7) resulting to similar environmental characteristics as sampled data being identified and projected over the study area [13]. We achieved this extrapolation using MAXENT algorithm with 68 occurrence points shown in Fig. 2a and environmental layers shown in Fig. 3, Fig. 4, Fig. 5, Fig. 6. 70% of the occurrence data were used to train the model while 30% was used for model evaluation. Fig. 1 shows the vector habitat suitability map generated with an accuracy of 0.75 Area Under Curve (AUC) of Receiver Operating Curve.

Fig. 1 also shows the home ranges for the collared herds. This was achieved by generating utilization distribution using Kernel Density Estimator (KDE) from the telemetry data shown in Fig. 2b [14]. The home range is defined as that area criss-crossed by an animal as part of its normal activity and movement due to food gathering, mating, and caring for the young [15]. Within given home ranges (Fig. 1), we have core areas that are frequently used by the animals than other areas [16]. The utilization distribution map describes this intensity of use within the home ranges using contour boundaries defining the space use percentage where 50% describes the ‘core area’ and 99% describes the entire home range [17]. The home range map was overlaid on vector habitat suitability map to identify risk zones in a GIS environment
